# Cryptic *Leishmania infantum *infection in Italian HIV infected patients

**DOI:** 10.1186/1471-2334-9-199

**Published:** 2009-12-10

**Authors:** Claudia Colomba, Laura Saporito, Fabrizio Vitale, Stefano Reale, Giustina Vitale, Alessandra Casuccio, Manlio Tolomeo, Daniela Maranto, Raffaella Rubino, Paola Di Carlo, Lucina Titone

**Affiliations:** 1 Dipartimento di Scienze per la Promozione della Salute, Sezione di Malattie Infettive, Università di Palermo, Palermo, Italy; 2 Centro di Referenza Nazionale per le Leishmaniosi (CReNaL), Istituto Zooprofilattico Sperimentale della Sicilia, Palermo, Italy; 3 Dipartimento di Medicina Clinica e delle Patologie Emergenti, Azienda Ospedaliera Universitaria Policlinico, Palermo, Italy; 4 Dipartimento di Neuroscienze Cliniche, Università di Palermo, Palermo, Italy

## Abstract

**Background:**

Visceral leishmaniasis (VL) is a protozoan diseases caused in Europe by *Leishmania (L.) infantum*. Asymptomatic *Leishmania *infection is more frequent than clinically apparent disease. Among HIV infected patients the risk of clinical VL is increased due to immunosuppression, which can reactivate a latent infection. The aims of our study were to assess the prevalence of asymptomatic *L. infantum *infection in HIV infected patients and to study a possible correlation between *Leishmania *parasitemia and HIV infection markers.

**Methods:**

One hundred and forty-five HIV infected patients were screened for the presence of anti-*Leishmania *antibodies and *L. infantum *DNA in peripheral blood. Statistical analysis was carried out by using a univariate regression analysis.

**Results:**

Antibodies to *L. infantum *were detected in 1.4% of patients. *L. infantum *DNA was detected in 16.5% of patients. Significant association for PCR-*Leishmania *levels with plasma viral load was documented (p = 0.0001).

**Conclusion:**

In our area a considerable proportion of HIV infected patients are asymptomatic carriers of *L. infantum *infection. A relationship between high HIV viral load and high parasitemic burden, possibly related to a higher risk of developing symptomatic disease, is suggested. PCR could be used for periodic screening of HIV patients to individuate those with higher risk of reactivation of *L. infantum *infection.

## Background

Leishmaniasis is a group of protozoan diseases, transmitted by the bite of a sandfly infected with *Leishmania *parasites, and including cutaneous, mucocutaneous and visceral manifestations. In Europe visceral leishmaniasis (VL) is caused by *Leishmania (L.)infantum *and is transmitted through a zoonotic mechanism which involves the dog as the main reservoir of the infection. It has been estimated that in endemic countries the number of asymptomatic *Leishmania *infections in immunocompetent persons is 5-10 times greater than the number of clinically apparent VL disease cases [1].

Much attention has been given to *Leishmania*/HIV coinfection during the last decade. Among patients with AIDS the risk of clinical VL is increased by 100-1000 times due to immunosuppression, which can reactivate a latent infection [2]. It is often mentioned in the literature that 25-70% of all VL cases in the Mediterranean countries are HIV-positive. Moreover, VL treatment is a challenge in these patients because of frequent relapses despite an adequate therapy. During the last years the incidence of VL in HIV infected patients in south western Europe is dramatically diminished due to the use of Highly Active Antiretroviral Therapy. Nevertheless, the carriage of *L. infantum *in peripheral blood has been proven in asymptomatic HIV-infected patients and in patients with an opportunistic infections other than VL [3,4].

Serological methods are useful for diagnosing VL in immunocompetent patients, but they always require a parasitological confirmation by direct detection of *Leishmania *amastigotes in bone marrow biopsy samples by microscopic observation, culture or polymerase chain reaction (PCR). Furthermore, serological tests are of limited value in HIV-associated VL, where their diagnostic sensitivity is much lower.

During the last years a number of noninvasive methods have been developed for the diagnosis of *Leishmania *infection. PCR-based methods for detecting *Leishmania *species have been used for testing peripheral blood and urine samples [5-7]. Particularly, PCR assays performed on peripheral blood samples have been confirmed as a useful tool for the diagnosis of VL in both immunocompetent and immunocompromised patients [5,8].

The aims of our study were to assess the prevalence of asymptomatic *L. infantum *infection in HIV infected patients living in our geographic area and to study a possible correlation between *Leishmania *parasitemia and HIV infection markers.

## Methods

One hundred and forty-five HIV infected patients attended at infectious disease department of Policlinico in Palermo (Italy) in the period February-May 2008 were invited to participate in a prevalence study. All 145 patients after giving their consent were screened for both the presence of anti-*Leishmania *antibodies and *L. infantum *DNA in peripheral blood. From all patients demographic and clinical data were collected. In particular, information regarding the previous occurrence of symptomatic VL were asked. The study was carried out in compliance with the Helsinki declaration.

### Serological analysis

Serum samples were analyzed for the presence of anti-*Leishmania *IgG antibodies by an immunofluorescent antibody test (IFAT) and an enzyme-linked immunosorbent assay (ELISA).

For IFAT, a laboratory-made antigen was used to increase test sensitivity [9]. Promastigotes of *L. infantum *zymodeme MON1 were propagated in Medium 199 (Gibco, Milan, Italy) supplemented with 25 mM Hepes (Gibco, Milan, Italy) and 10% fetal calf serum (Gibco, Milan, Italy). After 48 hours of incubation at 26°C, whole parasites were collected by centrifugation and washed three times in cold phosfate-buffered saline (PBS). For IFAT, whole parasite were fixed on slides (Bio-Merieux Italia, Rome, Italy), diluted in PBS until to obtain 30-40 parasites for microscopic field. For IFAT we used the fluorescein-conjugated anti-human immunoglobulin IgG (KPL Gaithersburg, USA). The optimal working concentration was 1:100 in PBS.

For ELISA, soluble leishmanial antigen was prepared by ultrasonic lysed and by cycles of freezing and thawing of a suspension of parasites. The optimal protein concentration was 0,2 μg/well, as previously reported [10]. We used for ELISA, anti-human IgG labelled with alkaline phosphatase (SIGMA- Aldrich srl, Milano, Italy); the optimal working concentration was 1:5000 in PBS. The cut-off points for IgG was defined as the arithmetical mean OD of sera of blood donors + 3 standard deviations (0.300).

Both for IFAT and ELISA, all samples showing positivity at a dilution ≥1/50 were considered to be reactive.

### DNA extraction

DNA was extracted from cultured IPT1 MON1 *Leishmania *promastigotes (to construct the standard curve), and from human blood samples. The IPT1, taken from the collection of the Italian National Reference Centre for leishmaniasis (C.Re.Na.L), were grown in Tobie agar medium, Evans modified [11,12]. Stationary-phase promastigotes were harvested by centrifugation, washed in NaCl 0.3%, and enumerated with a Thoma hemacytometer. Finally the pelleted parasites were resuspended in PBS at 1 × 10^9^/ml final concentration and employed for DNA extraction. The cells were again pelleted at 4.000 rpm for 15 min, and homogenized in 1 ml of lysis mix, (1% Tween 20, 1% Non idet P-40, e 20% Chelex). The mixture was incubated at 96°C for 20 min until complete lysis. After 10 min centrifugation at 14000 rpm we collected the liquid containing the extracted DNA. The examined human samples, were 200 μl of whole blood, that were extracted by "Illustra blood genomic Prep mini Spin kit (GE)" according to the manufacturer instruction.

### TaqMan PCR

The PCR test was targeted on a 68 bp fragment inner the constant region in the mini circle kinetoplast DNA (kDNA) (NCBI accession number AF291093) and was carried out as previously described [13]. The primers and probe were chosen with the assistance of *Primer express *software (Applied Biosystem). The primer sequences were: QLK2-U 5'-GGCGTTCTGCGAAAACCG-3'; QLK2-D5'-AAAATGGCATTTTCGGGCC-3'; while the associated probe was: 5'-TGGGTGCAGAAATCCCGTTCA-3' 5'FAM and 3' TAMRA labelled. We introduced a reaction quality internal control system supplied by the "*TaqMan exogenous internal positive control reagents kit*" (Applied Biosystem), labelled with a "VIC" fluorochrome. Each amplification was performed in duplicate, in 25 μl reaction mixture containing 1× TaqMan Universal Master Mix (Applied Biosystem), 100 pmol/μl of the specific primers and 10 pmol/μl of labelled probe (Qleish 2), 1× EXO IPC Mix, 1× EXO IPC DNA according to the manufacturer's instructions of the TaqMan Exogenus Internal Positive Control Reagents kit (Applied Biosystem). The thermal cycling conditions comprised an initial incubation for 2' at 50°C for uracyl-N-glycosylase activity. This step was followed by a 10' denaturation at 95°C and 45 cycles at 95°C for 15" and 60°C for 1' each. The quantity of DNA in the samples examined was detected by comparison of the Ct values plotted on common log scale.

Results were expressed as parasite charge for ml of the liquid matrices as blood, according to the parasite charge per ml of the standard curve described below. The efficiency of the amplification was always close to 1. Reproducibility was previously estimated by testing each dilution of the standard DNA for 18 times in each analytical plate. Three replicates of six different concentrations of *L. infantum *DNA were tested in the same run and the experiment was done four times, (72 test totally); in this way we performed intra-assay and inter-assay comparison of the obtained signal for each DNA concentrations. The reproducibility was always 100% for each analytical point of the standard curve ranging from the DNA equivalent of 1 × 10^6 ^cells to 1 cell for μl. We detected the limit of the sensitivity, by testing decimal serial dilutions of the standard DNA below to 1 parasite equivalent per ml. Reproducibility of the PCR *Leishmania *test was 100% at a level of DNA concentration corresponding to 0.1 parasites/ml, which was considered the threshold for positive samples.

### Standard curve

A standard stock DNA solution was obtained by extraction of 1 ml PBS containing 1 × 10^9 ^IPT1 MON1 promastigotes as described above. We performed decimal serial dilutions of the stock solution to obtain the points of the curve ranging from the DNA equivalent of 1 × 10^6 ^cells to 1 cell for μl. The difference for each point of the curve was one log factor. The DNA concentration was estimated by spectrophotometric determination of *A*_260 _and *A*_280. _and by gel electhrophoresis. On the basis of the linearity in the fluorescent signal trough the serial standard DNA dilutions, PCR test and dedicated software (SDS Applied Biosystems) permitted us to detect parasitic charge lower than 1 cell/ml (minimum of the curve).

### Statistical analysis

Data were expressed as median and range unless otherwise specified. Linear regression analysis examined the correlation between CD4 count, plasma viral load (independent variables), and *L. infantum *kDNA levels (dependent variable) in the HIV infected patients. These continuous variables were assessed in univariate linear regression model. All P-values were two-sided and P-values less than 0.05 were considered statistically significant. Data were analyzed by the Epi Info software (version 6.0, Centers for Disease Control and Prevention, Atlanta, GA, USA) and SPSS Software (version 14.0, SPSS Inc, Chicago, IL, USA).

## Results

The study population comprised 145 patients, 99 males and 46 females. The median age was 43 years (range 22-70 years). One hundred and thirty-three blood samples were obtained from asymptomatic patients who consecutively referred to our Infectious Diseases ambulatory. Twelve blood samples belonged to HIV infected patients admitted to our Infectious Diseases department with symptoms not consistent with VL: 5 patients were admitted for pneumopathy, 2 for endocarditis, 2 for urinary tract infection, 1 for tuberculosis, 1 for Kaposi's sarcoma and 1 for neurotoxoplasmosis. Median CD4 cells count was 430 cells/mm^3 ^(range 7-1264). Viral load was undetectable (<47 HIV RNA copies/ml) in 52 patients. One hundred and fourteen patients assumed antiretroviral therapy. Only one patient reported a previous diagnosis of VL 5 years before, that was successfully treated with liposomal amphotericin B.

Specific IgG antibodies to *L. infantum *were detected both by ELISA and IFAT method in 2/145 (1.4%) patients.

*L. infantum *kDNA was detected by PCR in 24/145 (16.5%) asymptomatic patients. Parasitemia was detected also in one additional patient, who had been admitted for neurotoxoplasmosis, and during the hospitalization developed severe anemia and splenomegaly. For this reason he was treated with intravenous liposomal amphotericin B and was excluded by analysis.

**Figure 1:**
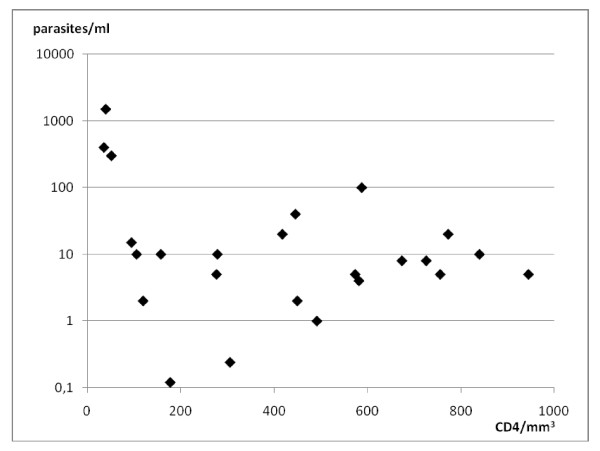
**Distribution of *Leishmania infantum *parasitemias related to CD4 cells count in 24 HIV infected patients, asymptomatic for *L. infantum *infection**.

**Figure 2:**
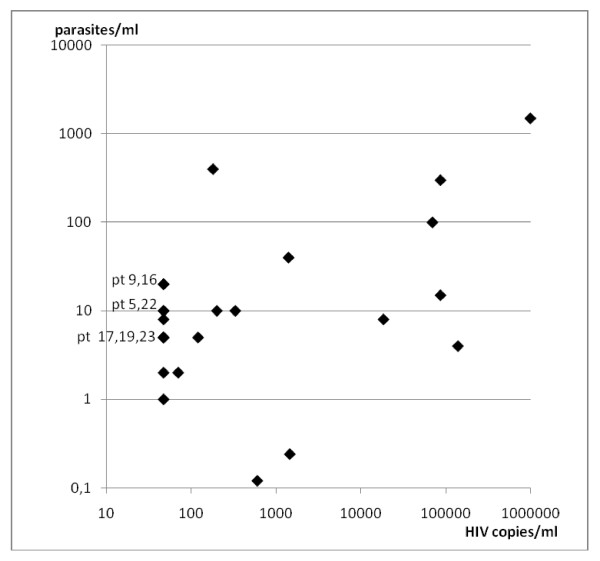
**Distribution of *Leishmania infantum *parasitemias related to HIV viral load in 24 HIV infected patients, asymptomatic for *L. infantum *infection**. pt = patients.

Parasitemia ranged between 0.12 and 1500 parasites/ml with a median of 9 parasites/ml (table 1). Median CD4 cell count was 432 cells/mm^3 ^(range 36-945). HIV viral load ranged between <47 and >1000000 copies/ml with a median of 150 copies/ml (figures 1, 2). All parasitemic patients but 2 (patients 12, 13) were HIV infected outpatients attending our Infectious Diseases ambulatory. Patient 12 was affected by urinary tract infection; patient 13 was affected by pneumopathy.

**Table 1:** Characteristics of 24 HIV infected patients with asymptomatic *Leishmania infantum *parasitemia

**Pt no**.	Sex	Age	Previous VL	CD4 (cells/mm^**3**^)	Viral load (copies/ml)	ELISA	IFAT	PCR (parasites/ml)
1	m	39	No	492	<47	neg	Neg	1

2	m	32	No	306	1450	neg	Neg	0.24

3	m	46	No	450	70	neg	Neg	2

4	f	42	No	178	600	neg	Neg	0.12

5	m	43	No	840	<47	neg	Neg	10

6	f	37	No	106	200	neg	Neg	10

7	m	42	No	588	70000	neg	Neg	100

8	f	33	No	446	1400	neg	Neg	40

9	f	48	No	773	<47	neg	Neg	20

10	m	33	No	95	87000	neg	Neg	15

11	m	45	No	158	330	neg	Neg	10

12	m	53	No	277	120	neg	Neg	5

13	m	37	No	52	87000	neg	Neg	300

14	m	50	2003	40	>1000000	1:400	1:1600	1500

15	m	29	No	36	180	neg	Neg	400

16	f	43	No	418	<47	neg	Neg	20

17	m	43	No	574	<47	neg	Neg	5

18	m	41	No	120	<47	1:400	1:800	2

19	m	42	No	756	<47	neg	Neg	5

20	f	48	No	674	18500	neg	Neg	8

21	f	43	No	582	140000	neg	Neg	4

22	m	49	No	279	<47	neg	Neg	10

23	m	54	No	945	<47	neg	Neg	5

24	f	53	No	726	<47	neg	Neg	8

We performed univariate linear regression analysis to estimate the association of *L. infantum *kDNA levels (dependent variable) with the number of CD4 cells and plasma viral load (independent variables) in the HIV infected patients. Univariate regression analysis showed a significant association for PCR levels with plasma viral load (p = 0.0001) but not with CD4 cells (p = 0.061) (table 2).

**Table 2:** Univariate linear regression analysis

	Univariate analysis		
**Independent variable**	**Slope Coefficient (SE)**	**Coefficient of determination R**^**2**^	**P**

CD4 cell count	- 0.431(0.218)	0.151	0.061

Viral load	0.0014(0.0001)	0.893	0.0001

Dependent variable = *L. infantum *kDNA levels; SE = standard error

Patients who tested positive for *L. infantum *kDNA were tested again on the subsequent ambulatory visit. Until now, six patients have been retested. In five of them, PCR was negative and CD4 cells count was higher than the first determination, or almost the same; the last patient had an increased parasitemia level accompanied by a reduced CD4 cells count.

## Discussion

VL is a relatively common disease in Europe, its incidence rates varying from 0.3 to 5.6/100000/year in the endemic countries [14]. Italy accounts for a mean of 155 cases/years with an incidence rate of 0.3/100000.

In the present study, specific IgG were detected only in 2/24 (8.3%) PCR-positive patients. Currently, these patients have neither fever, splenomegaly nor pancytopenia. The first patient (table 1, patient 14) had been previously cured for VL in 2003. The high parasitemia detected in this patient could be caused by the recent worsening of his CD4 cells count, so he is strictly monitored to survey a possible reactivation of VL. The second patient (table 1, patient 18) had no history of previous VL, and his parasitemia is very low, so the risk of a symptomatic VL is probably low. Nevertheless, he will be monitored even if his HIV infection seems to be quite controlled. As previously reported, there is no strict concordance between serological and parasitological techniques for the detection of cryptic *L. infantum *infection [15,16]. Moreover serological tests have been described as limited diagnostic tools in HIV infected people, since only 40% of VL patients appeared seropositive for leishmaniasis. This fact is probably due to the pronounced disregulation of the immune system that occurs in HIV infection [2,4].

Since immunosuppressed patients present a higher risk of developing overt disease than immunocompetent people, it is likely that the number of asymptomatic carriers of *L. infantum *among HIV infected persons would be higher as well. Our results highlight that in our geographic area a considerable proportion (16.5%) of HIV infected patients are asymptomatic carriers of *L. infantum *infection. A similar screening performed in Spain found *L. infantum *DNA in peripheral blood in 30.4% of 92 asymptomatic HIV infected subjects [4]. The lower percentage of parasitemia detected in our study could be attributable to a lower prevalence of the asymptomatic leishmaniasis in our endemic area. This is in accordance with the low incidence of reported cases of VL in Italy, which is one of European countries with a slight endemicity. Furthermore, recent studies carried out in our region showed a low prevalence of seropositivity for *L. infantum *in blood donors [17,18].

The cell-mediated immune response largely determines the outcome of *Leishmania *infection, and therefore most cases of overt VL in HIV patients occur when CD4 cells count is under 200/mm^3 ^[2,5,19]. Accordingly, while several studies performed in HIV infected patients with VL reported a median CD4 cells count lower than 100 cells/mm^3 ^[5,19], our study together with previous observations shows that *L. infantum *infection can be asymptomatic in HIV patients probably thanks to higher CD4 cells counts [3,4]. The correlation between HIV-viral load and parasitemia documented by our study (table 2) underlines the synergism between *Leishmania *and HIV.

Real- time PCR allows to quantify the number of parasites circulating in peripheral blood. It has been reported that the parasite burden in patients with VL and concomitant HIV infection is similar to that found in immunocompetent patients with VL [5,19]. However, we observed that among HIV asymptomatic patients the median parasite burden is lower than that detected in HIV patients with VL [19]. This is consistent with our finding of a significant association for PCR levels with plasma viral load (table 2), and underlines the relationship between HIV and *Leishmania *infections. In fact, those asymptomatic patients with the higher PCR values also had high HIV viral load (table 1). It is important to notice that the higher risk to develop symptomatic VL which probably concerns these patients would have not been recognized if the screening for *Leishmania *infection would have been performed only by serological analysis. These patients will undergo a more strict survey towards the appearance of clinical and/or laboratory signs of a reactivation of *Leishmania *infection. Until now none of parasitemic patients developed symptomatic VL.

Finally, the median parasitemia in our group of HIV infected patients resulted higher than that previously reported in healthy subjects with cryptic *Leishmania *infection living in endemic areas [15]. This evidence is likely due to immunological impairment, leading to a transitory reactivation of cryptic *Leishmania *infection resulting in spikes of parasitemia higher than those detected in subjects with asymptomatic *Leishmania *infection without HIV coinfection.

## Conclusion

In conclusion our findings seems to suggest a relationship between high HIV viral load and high parasitemic burden in HIV infected patients with cryptic *L. infantum *infection. Hence, as showed by regression analysis, the plasma viral load could be considered a good independent marker of a increase in the PCR parasitemia levels. This association could be related to a higher risk of developing symptomatic disease. However, further studies are needed to assess the possible correlation between *Leishmania *parasitemia and HIV infection markers because the wide variability both in viral load and in parasitemia values, together with the relatively small number of patients, make difficult to establish a definitive relationship. Real-time PCR could be considered a rapid and useful tool for periodic screening of HIV infected asymptomatic patients, in order to individuate those patients with higher risk of reactivation of *Leishmania *infection and to survey the long term outcomes of asymptomatic infections by *L. infantum *in HIV infected patients.

## Competing interests

The authors declare that they have no competing interests.

## Authors' contributions

CC and LS ideated and designed the study and drafted the manuscript. AC performed the statistical analysis. PD participated in the design of the study. DM and RR were involved in the collection of blood samples and in the acquisition of clinical data. GV and MT carried out the serological tests. FV and SR carried out the molecular analysis and helped to draft the manuscript. LT participated in the design and coordination of the study and revised it critically. All authors participated in the analysis and interpretation of data, and read and approved the final manuscript.

## Pre-publication history

The pre-publication history for this paper can be accessed here:

http://www.biomedcentral.com/1471-2334/9/199/prepub
